# The National Cancer Database Conforms to the Standardized Framework for Registry and Data Quality

**DOI:** 10.1245/s10434-024-15393-8

**Published:** 2024-05-08

**Authors:** Bryan E. Palis, Lauren M. Janczewski, Amanda E. Browner, Joseph Cotler, Leticia Nogueira, Lisa C. Richardson, Vicki Benard, Reda J. Wilson, Nadine Walker, Ryan M. McCabe, Daniel J. Boffa, Heidi Nelson

**Affiliations:** 1 American College of Surgeons, Chicago, IL USA; 2 American Cancer Society, Atlanta, USA; 3 Centers for Disease Control and Prevention, Atlanta, USA; 4 National Cancer Registrars Association, Alexandria, USA; 5 Yale School of Medicine, New Haven, USA; 6 Department of Surgery, Mayo Clinic, Rochester, USA

**Keywords:** NCDB, Registry, Quality, Standardization, Coverage, Comparability, Timeliness, Validity

## Abstract

**Background:**

Standardization of procedures for data abstraction by cancer registries is fundamental for cancer surveillance, clinical and policy decision-making, hospital benchmarking, and research efforts. The objective of the current study was to evaluate adherence to the four components (completeness, comparability, timeliness, and validity) defined by Bray and Parkin that determine registries’ ability to carry out these activities to the hospital-based National Cancer Database (NCDB).

**Methods:**

Tbis study used data from U.S. Cancer Statistics, the official federal cancer statistics and joint effort between the Centers for Disease Control and Prevention (CDC) and the National Cancer Institute (NCI), which includes data from National Program of Cancer Registries (NPCR) and Surveillance, Epidemiology, and End Results (SEER) to evaluate NCDB completeness between 2016 and 2020. The study evaluated comparability of case identification and coding procedures. It used Commission on Cancer (CoC) standards from 2022 to assess timeliness and validity.

**Results:**

Completeness was demonstrated with a total of 6,828,507 cases identified within the NCDB, representing 73.7% of all cancer cases nationwide. Comparability was followed using standardized and international guidelines on coding and classification procedures. For timeliness, hospital compliance with timely data submission was 92.7%. Validity criteria for re-abstracting, recording, and reliability procedures across hospitals demonstrated 94.2% compliance. Additionally, data validity was shown by a 99.1% compliance with histologic verification standards, a 93.6% assessment of pathologic synoptic reporting, and a 99.1% internal consistency of staff credentials.

**Conclusion:**

The NCDB is characterized by a high level of case completeness and comparability with uniform standards for data collection, and by hospitals with high compliance, timely data submission, and high rates of compliance with validity standards for registry and data quality evaluation.

Medical practices and advances in health care are information dependent, and both rely on high-quality data. In recent years, the availability of health care data and analytic platforms has grown exponentially with increasing use of electronic medical records and insurance claims. However, just as the evidence generated by clinical trials is rigorously tested through a set of preexisting data quality procedures,^1,2^ other sources of data also could be graded in a uniformly defined and regulated manner.

The usability of all data sources is crucial to understanding strengths and limitations. With new data sources becoming more accessible among clinicians and researchers to help shape the future of health care, ensuring data quality through a standardized evaluation plays an increasingly critical role. One such standardized approach to assessing the quality of data collected by cancer registries is the framework described by Bray and Parkin^3,4^ in 2009.

The Bray and Parkin registry and data quality framework was developed with four unique domains: completeness, comparability, timeliness, and validity.^3,4^ Completeness represents the extent to which all the incidences of cancer occurring in the population are included in a registry.^3,4^ Completeness is crucial for ensuring that estimates approximate the true value in the population.^3,4^ Comparability represents the extent to which statistics generated for different populations, using data from different sources and over time, can be compared.^3,4^ Comparability is achieved using standardized guidelines on classification procedures, maintaining consistency for coding cancer cases.^3,4^ Timeliness relates to the rapidity through which a registry can abstract and report reliable cancer data, which is crucial for decision-making.^3,4^ Validity represents the proportion of cases in a dataset with a given characteristic that truly has that attribute, which is crucial for relevant interpretation of estimates calculated using the data.^3,4^ Importantly, this framework has been applied across numerous cancer registries worldwide, demonstrating its ability to affirm, document, and benchmark data quality.^5–7^

The processes that ensure data quality of both population- and hospital-based cancer registries in the United States of America (USA) have been consistent for several decades and include standardization of data-field definitions, quality checks executed during data abstraction, and case monitoring after submission (Fig. 1). The principal aim of a population-based cancer registry is to record all new cases in a geographic area or state, with an emphasis on epidemiology and public health.^8,9^ By contrast, a hospital-based registry is designed to improve patient quality of care at the institutional level.^8,9^ Both population- and hospital-based cancer registries adhere to uniform procedures during the record abstraction and coding process to ensure accuracy but serve different purposes.

**Fig. 1:**
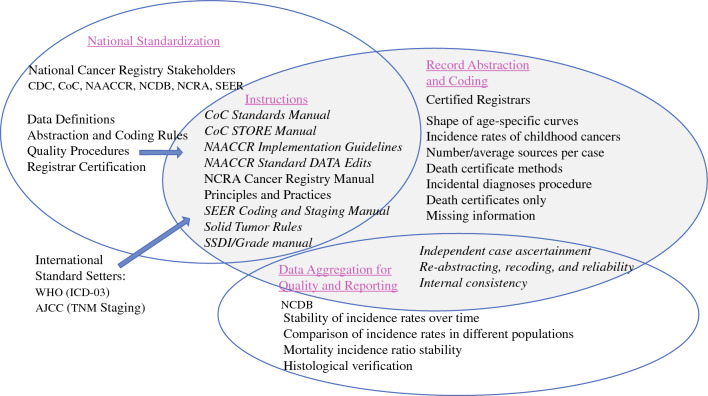
National Cancer Registry quality processes. The quality of cancer data in the United States is supported by a large, multi-agency, National Cancer Registry stakeholder community in the United States that works collaboratively to ensure consistent, high-quality cancer data that can be applied across diverse utilities. These National Cancer Registry stakeholders standardize cancer data definitions, abstraction and coding rules, and registry-based quality procedures as well as registrar education, training, and certification. These national standards are monitored at the hospital level through compliance with quality procedures during the record abstraction and coding process as well at the national level during the process of data aggregation for quality and reporting. *AJCC* American Joint Committee on Cancer, *CDC* Centers for Disease Control and Prevention, *CoC* Commission on Cancer, *NAACCR* North American Association of Central Registries, Inc.; *NCDB* National Cancer Data Base, *NCRA* National Cancer Registrars Association, *SEER* Surveillance, Epidemiology, and End Results Program, *STORE* Standards for Oncology Registry Entry, *SSDI* Site-Specific Data Item, *WHO* World Health Organization

The reporting of cancer cases to the population-based central cancer registry (CCR) is mandated by legislation in the USA and territories.^10,11^ The cases identified by these CCRs are then reported to national cancer registries.^10–12^ The reporting of cancer cases within a hospital is mandated by the hospital-based National Cancer Database (NCDB) to maintain accreditation from the Commission on Cancer (CoC).^13,14^ Although the Bray and Parkin quality control criteria were written primarily with population-based registries in mind, we propose their use for large hospital-based registries, such as the NCDB.

Cancer surveillance programs collaborate to standardize definitions of relevant cancer data items and closely monitor estimates of cancer trends and outcomes calculated using different data sources.^9^ Each cancer surveillance program works with oncology data specialist (ODS)-certified cancer registrars who are educated, trained, and certified in abstracting cancer data following established definitions and rules.^9,15^ Although these processes, among many others, have demonstrated consistency over time, they also are dynamic and undergo periodic revisions to incorporate advances in cancer care and ensure the availability of contemporary cancer data.^9,15^

The NCDB is a hospital-based cancer registry and contains approximately 40 million records, collecting data on patients with cancer since 1989.^16,17^ The NCDB is jointly maintained by the American College of Surgeons CoC and the American Cancer Society.^13,17^ To earn voluntary CoC accreditation, a hospital must meet quality of patient care and data quality standards.^13^ Hospitals are evaluated on their compliance with the CoC standards on a triennial basis through a site visit process to maintain levels of excellence in the delivery of comprehensive patient-centered care.^13^ The CoC standards are designed to ensure that the processes of the hospital’s cancer program support multidisciplinary patient-centered care.^13^ Adherence to these standards is required to maintain accreditation in the CoC. The standards demonstrate a hospital’s investment in structure along a full continuum from cancer prevention to survivorship.^13^

Overall, approximately 1500 CoC-accredited hospitals submit data to the NCDB each year.^16^ The NCDB collects data from patients in all phases of first-course treatment in cancer care and cancer surveillance and includes the addition of roughly 1.5 million records with newly diagnosed cancers annually.^14,16,17^ Reportable cancer diagnoses will originate from single- and multi-institution cancer registries.^18^ The fundamental purpose of the NCDB is to capture data designed to improve patient outcomes.^18^

Evidence-based quality measures representing clinical best practice are reported from the NCDB through interactive benchmarking reports.^13^ This includes the Rapid Cancer Reporting System (RCRS), a web-based tool designed to facilitate real-time reporting of cancer cases.^13^

Although registrars who submit data to the NCDB are involved in aspects of both the population-based registries and the hospital-based registries, not all quality procedures performed by registrars pertain to the NCDB (Table 1). Quality procedures identified by Bray and Parkin that are relevant only to population-based cancer registries include assessment of age-specific curves, incidence rates of childhood cancers, mortality incidence ratio stability, number and average sources per case, and death certificate methods.^11^ Death certificate-only analyses are performed routinely across all population-based registries.^11^ Death certificate analysis as a quality indicator does not directly affect the NCDB. However other quality procedures are performed after data submission as part of data aggregation, quality assessment, and reporting within the NCDB.

**Table 1:** Assessment of NCDB registry and data quality according to Bray and Parkin criteria

Bray and Parkin criteria		Application to NCDB^a^	Data quality mechanism	Registry and data quality category
*Completeness*				
Historic data methods	Stability of incidence rates over time	Yes	Annual NCDB warehouse quality assurance check; annual benchmarking report trends	Quality procedure
	Comparison of incidence rates in different populations	Yes	NCDB coverage to USCS	Quality procedure
	Shape of age-specific curves	No	*NAACCR Standards for Cancer Registries Volume III* (age-specific/adjusted incidence rates)	Quality procedure
	Incidence rates of childhood cancers	No	*NAACCR Standards for Cancer Registries Volume III* (percent incidence ratio)	Quality procedure
Mortality incidence ratio stability		No	*NAACCR Standards for Cancer Registries Volume III*	Quality procedure
Number/average sources per case		No	*NAACCR Standards for Cancer Registries Volume III* (sources for reporting)	Quality procedure
Histologic verification		Yes	*NAACCR Standards for Cancer Registries Volume III*	Quality procedure
Independent case ascertainment		Yes	CoC special studies *NAACCR Standards for Cancer Registries Volume III* (NAACCR abstraction and recoding reliability studies and audits) *NCRA Cancer Registry Manual Principles and Practices,* 4th edition	Quality procedure
Death certificate methods		No	*NAACCR Standards for Cancer Registries Volume III* (percent death certificate only) *NAACCR Standards for Cancer Registries Volume III* (death clearance follow-back)	Quality procedure
*Comparability*				
Identification	Topography	Yes	WHO ICD-O-3 (C00.0-80.9)	Standardized data definition
	Histology	Yes	WHO ICD-O-3 (8000-9993)	
	Behavior	Yes	WHO ICD-O-3 (0-3)	
	Grade	Yes	NAACCR SSDI/*Grade Manual* WHO ICD-O-3	
Stage		Yes	AJCC staging standards	Standardized data definition
Secondary diagnosis		Yes	WHO ICD-10	Standardized data definition
Standard coding schema		Yes	*CoC STORE Manual* NAACCR SSDI/*Grade Manual* *NAACCR Data Standards and Data Dictionary* *SEER Coding and Staging Manual* 2023 SEER drug database	Abstraction and coding rules
Definition of incidence (case and date)		Yes	*NAACCR Standards for Cancer Registries Volume III* (diagnostic confirmation, class of case, type of submission, ambiguous terminology) CoC store manual	Abstraction and coding rules
Primary cancer (new case) rules		Yes	Solid tumor rules (collaborative product of CDC, NAACCR, SEER, and central registries)	Abstraction and coding rules
*Timeliness*				
Abstraction and submission timeliness		Yes	*NAACCR Standards for Cancer Registries Volume III* *CoC Standards Manual* 6.4	Abstraction and coding rules
Validity				
Re-abstracting, recoding, and reliability		Yes	*CoC Standards Manual 6.1* (review of 10 % analytic caseload annually) *NAACCR Standards for Cancer Registries Volume III* (QA process controls, special assessments, re-abstraction audits, recoding audits, reliability studies)	Quality procedure
Histologic verification		Yes	*CoC Standards Manuals 3.2 and 5.1* (accreditation for anatomic pathology, internal audit of 90 % of pathology reports annually)	Quality procedure
Death certificate only		No	*NAACCR Standards for Cancer Registries Volume III* (DCO validity) *SEER Coding and Staging Manual,* 2023	Quality procedure
Reviews’ missing information		Yes	*NAACCR Standards for Cancer Registries Volume III* (edits, process controls for unknown values) *Requirements NAACCR Standard Edits for Cancer Registry Volume IV*	Quality procedure
Reviews’ internal consistency		Yes	*CoC Standards Manual 4.3* (cancer registry staff credentials) *NAACCR Standards for Cancer Registries Volume III* (quality assurance standards, staffing guidelines, procedures, staff credentials) *NCRA Cancer Registry Manual Principles and Practices,* 4th ed V22B and V23B NCDB/RCRS edits and submission	Quality procedure

*NCDB* National Cancer Database, *USCS* United States Cancer Statistics, *NAACCR* North American Association of Central Registries, Inc.; *CoC* Commission on Cancer, *NCRA* National Cancer Registrars Association, WHO World Health Organization, *ICD* International Classification of Diseases, *SSDI* Site-Specific Data Item, *AJCC* American Joint Committee on Cancer, *STORE* Standards for Oncology Registry Entry, *SEER* Surveillance, Epidemiology, and End Results Program, *CDC* Centers for Disease Control and Prevention, *RCRS* Rapid Cancer Reporting System

^a^Procedures followed by all registrars for purposes of reporting to population-based registries that may not have a direct impact on reporting to the NCDB

The NCDB is part of a multi-agency, National Cancer Registry community in the USA that works collaboratively to ensure that consistent, high-quality cancer data can be applied across diverse utilities (Fig. 1). This surveillance community comprises the central cancer registries, including the Centers for Disease Control and Prevention (CDC), National Program of Cancer Registries (NPCR), and the Surveillance, Epidemiology, and End Results (SEER) Program of the National Cancer Institute (NCI); the National Cancer Registrars Association (NCRA); and the CoC.^19^ The North American Association of Central Cancer Registries (NAACCR) is also part of this community and serves a vital role as a consensus organization.^11^ The NAACCR facilitates standardization of data definitions, abstraction and coding rules, quality procedures, and registry certification, which in turn ensures uniform registry processes and establishes data quality standards.^11^ Instructions to support standardized data definitions, abstraction, and coding rules, as well as quality procedures, are detailed in key manuals and documents.^11^

An assessment of existing quality processes and procedures is fundamentally important to ensuring that the best possible data are being used to inform cancer practices and policies. The principal aim of this study was to assess the quality of cancer data collected by the NCDB using the Bray and Parkin framework.

## Methods

### Completeness

Completeness, defined as a measure of representation, is the extent to which all the incident cancer cases occurring in the population are included in the registry. Case-finding procedures are considered critical to both cancer registry coverage and survival accuracy. Completeness includes nine quality procedures (Table 1).^3,4^

Because of the legislative mandate to report cancer cases to population-based cancer registries in the USA, population-based cancer registries are regarded as the gold standard for data completeness.^11^ We evaluated data completeness within the NCDB by comparing the number of incident cancer cases from participating central registries included in the United States Cancer Statistics (USCS), the official federal cancer statistics.^12^ These statistics include cancer registry data from the CDC’s NPCR and the NCI SEER program.^12^ The USCS internal quality control file includes cases from all 50 states and the District of Columbia, providing information on demographic and tumor characteristics.^12^

Cancers diagnosed at a Veterans Affairs hospital were excluded from the NCDB analysis. Cases were further limited to malignant disease except for benign and borderline brain and other nervous system cancers and female in situ breast cancers. Only male and female cancers diagnosed within the USA between 2016 and 2020 were included.

The percentage of cancer cases captured within the NCDB from 2016 to 2020 were compared against prior reports, which included diagnostic years 2012 to 2014.^14^ Comparisons were made by primary disease site using the SEER definitions of the World Health Organization (WHO) International Classification of Diseases for Oncology, third-edition (ICD-O-3) site recodes.^20^ Additional stratification included sex, diagnosis year, patient age, race/ethnicity, and state of diagnosis corresponding to the patient’s residence.

Outcomes for other measures of completeness that affect all registries (Table 1) have been previously reported.^21^ Incidence case ascertainment for the NCDB is continuously verified with CoC special studies, which are required for accreditation, and specifically capture additional data on previously submitted cancer diagnoses. This provides an extra level of detail and audit of abstraction accuracy. Independent studies using data from the NCDB have demonstrated case ascertainment compared with trials and claims data.^22–24^ This type of auditing may be extended to assess registry completeness.

### Comparability

The study ensured comparability by using standardized international guidelines on coding and classification procedures for cancer data abstraction.^3,4^ Cancers reported to the NCDB are identified by the WHO ICD-O-3 topography, morphology, behavior, and grade codes.^25^ The ICD-O-3 and topography and histology codes are categorized into cancer types.^15,26–28^ Coding rules are maintained in registry manuals so that data items are abstracted and submitted to the registry with universal rules and codes.^15,26–28^ Staging standards are defined by the American Joint Committee on Cancer (AJCC).^29^ The rules for coding include timing relative to initiation of treatment. Clinical staging includes the extent of cancer information before initiation of definitive treatment or within 4 months after the date of diagnosis, whichever is shorter.^29,30^ Pathologic staging includes any information obtained about the extent of cancer through completion of definitive surgery or within 4 months after the date of diagnosis, whichever is longer.^29,30^ Secondary diagnosis codes are captured by the cancer registry as International Classification of Diseases, 10th Revision codes.^30^ The CoC also requires registries to submit up to 10 comorbid conditions to the NCDB. These conditions influence the health status of the patient and treatment complications.^30^

An interactive drug database maintained by SEER facilitates the proper coding of treatment fields.^31^ The rules for diagnostic confirmation require the reportability of both clinically diagnosed and microscopically confirmed tumors.^30^ Clinically diagnosed tumors are those with the diagnosis based only on diagnostic imaging, laboratory tests, or other clinical examinations, whereas microscopically confirmed tumors include all tumors with positive histopathology.^11,30^ Cancer registries reference both “ambiguous terms at diagnosis” to determine case reportability and “ambiguous terms describing tumor spread” for staging purposes.^30^ For reportability, the NCDB follows rules for class of case to describe the patient’s relationship to the facility. Rules exist for the reporting of multiple primary tumors to the NCDB.^32^ These solid tumor rules are aimed at promoting consistent and standardized coding by cancer registrars and are intended to guide registrars through the process of determining the correct number of primary tumors.^32^

### Timeliness

No international guidelines for cancer registry data submission timeliness exist, although the cancer surveillance community has specific timeliness standards for their respective registries.^11^ Timeliness of NCDB data submission was assessed using compliance with CoC standard 6.4 (Table 1).^13^

### Validity

Validity is defined by Bray and Parkin^3,4^ as the proportion of cases in a dataset with a given characteristic that has this characteristic. Data validity is maintained through procedures specific to quality control that are integral to the registry and tied to CoC standards 3.2, 4.3, 5.1, and 6.1 for CoC accreditation (Table 1).^13^

Accreditation for anatomic pathology by a qualifying organization is a component of standard 3.2, designed to further structure quality assurance protocols.^13^ Histologic verification also is assessed in compliance with CoC standard 3.2 and ensures that each hospital provides diagnostic imaging services, radiation oncology services, and systemic therapy services on site with accreditation by a qualifying organization for anatomic pathology.^13^

Compliance with CoC standard 4.3 is assessed for internal consistency, which ensures that all case abstraction is performed by cancer registrars who hold current certification by the NCRA.^13,15^ This ensures that registrars use, maintain, and continue their formal education through NCRA and thus continue working toward correct interpretation and coding of cancer diagnoses.^13,15^

Standard 5.1 requires College of American Pathologists^33^ synoptic reporting and for each hospital to perform an annual internal audit, confirming that at least 90 % of all cancer pathology reports are in synoptic format.^13^

The database validity criteria for re-abstracting, recoding, and reliability procedures identified by Bray and Parkin are measured in compliance with CoC standard 6.1. Additionally, data edits are integrated to maintain quality control.^11^ These electronic logical rules evaluate internal consistency of values or data items.^11^ For instance, a biologic woman with a diagnosis of prostate cancer will fail edits. Edits are currently maintained by NAACCR based on edits originally developed by SEER.^34^ The NAACCR Edits’ Metafile comprises validation checks applied to cancer data.^34^ The CDC develops and maintains software (EditWriter and GenEDITS Plus) for registries to obtain edit reports on their cases using the standards maintained by NAACCR.^34,35^ The NCDB assigns scores that are applied to the call for data and to RCRS reporting requirements, causing a case to be rejected or accepted into either dataset.^36^ An edit score of 200 will cause a record to be rejected from the NCDB.^36^

All data were analyzed using SAS version 9.4 (SAS Institute, Cary, NC, USA)^37^ or SEER Surveillance Research Program, National Cancer Institute SEER*Stat software version 8.4.2.^38^

## Results

The exclusion and inclusion criteria resulted in 9,269,442 cases from the USCS and 6,828,507 cases from the NCDB. Compared with the USCS, the official cancer statistics,^39^ the NCDB demonstrated 73.7 % completeness of cancer cases diagnosed in the USA between 2016 and 2020 (Table 2). Among the top 10 major cancer sites, breast cancer in males and females had the highest coverage, at 81.9%, and the lowest coverage was found for melanoma of the skin in males and females, at 52.0% (Table 2). In aggregate, coverage steadily increased from 73.0% in 2016 to 74.3% in 2020 (Table 3). Age group comparisons showed the lowest coverage (61.1%) for the patients 85 years of age or older, with the highest coverage for those 20–74 years of age (73.1–80.4%) (Table 3). Race and ethnicity comparisons showed coverage to be 68.4% for white patients, 73.7% for black patients, 41.0% for American Indian/Alaskan Native patients, 70.7% for Asian/Pacific Islander patients, and 56.4% for Hispanic patients (Table 3). Finally, by state, Arkansas demonstrated the lowest coverage (24.0%), and North Dakota demonstrated the highest coverage (98.9%) (Table 4).

**Table 2:** Comparison of incidence for completeness by disease sites in 2016–2020

	USCS Count	NCDB Count	Coverage (%)	USCS count (males)	NCDB count (males)	Coverage (%)	USCS count (female)	NCDB count (females)	Coverage (%)
Total^a^	9,269,442	6,828,507	73.7	4,522,387	3,142,113	69.5	4,747,055	3,686,394	77.7
Oral cavity and pharynx	239,509	188,806	78.8	171,188	134,296	78.4	68,321	54,510	79.8
Lip	9231	5192	56.2	6529	3724	57.0	2702	1468	54.3
Tongue	77,495	62,151	80.2	55,989	44,717	79.9	21,506	17,434	81.1
Salivary gland	24,196	18,421	76.1	14,148	10,414	73.6	10,048	8007	79.7
Floor of mouth	9601	8144	84.8	6473	5454	84.3	3128	2690	86.0
Gum and other mouth	31,711	26,133	82.4	17,751	14,617	82.3	13,960	11,516	82.5
Nasopharynx	9606	7278	75.8	6808	5123	75.2	2798	2155	77.0
Tonsil	46,527	37,638	80.9	38,815	31,401	80.9	7712	6237	80.9
Oropharynx	15,298	12,401	81.1	12,230	9902	81.0	3068	2499	81.5
Hypopharynx	11,255	9217	81.9	8949	7286	81.4	2306	1931	83.7
Other oral cavity and pharynx	4589	2231	48.6	3496	1658	47.4	1093	573	52.4
Digestive system	1,549,130	1,169,589	75.5	867,417	651,185	75.1	681,713	518,404	76.0
Esophagus	92,634	71,329	77.0	73,239	56,134	76.6	19,395	15,195	78.3
Stomach	122,455	92,974	75.9	75,013	57,554	76.7	47,442	35,420	74.7
Small intestine	49,807	39,377	79.1	26,716	21,047	78.8	23,091	18,330	79.4
Colon and rectum	711,415	527,686	74.2	375,758	277,230	73.8	335,657	250,456	74.6
Colon excluding rectum	502,914	366,984	73.0	252,651	182,490	72.2	250,263	184,494	73.7
Rectum and rectosigmoid junction	208,501	160,702	77.1	123,107	94,740	77.0	85,394	65,962	77.2
Anus, anal canal, and anorectum	39,893	32,411	81.2	13,778	11,061	80.3	26,115	21,350	81.8
Liver and intrahepatic bile duct	179,172	131,386	73.3	126,466	92,104	72.8	52,706	39,282	74.5
Gallbladder	21,348	16,380	76.7	7009	5305	75.7	14,339	11,075	77.2
Other biliary	33,101	28,506	86.1	18,280	15,814	86.5	14,821	12,692	85.6
Pancreas	267,894	204,543	76.4	139,094	105,999	76.2	128,800	98,544	76.5
Retroperitoneum	7771	6859	88.3	3968	3448	86.9	3803	3411	89.7
Peritoneum, omentum and mesentery	9430	8398	89.1	866	669	77.3	8564	7729	90.2
Other digestive organs	14,210	9740	68.5	7230	4820	66.7	6980	4920	70.5
Respiratory system	1,189,661	903,630	76.0	627,383	467,371	74.5	562,278	436,259	77.6
Nose, nasal cavity, and middle ear	12,771	11,010	86.2	7795	6691	85.8	4976	4319	86.8
Larynx	61,328	47,936	78.2	48,699	37,680	77.4	12,629	10,256	81.2
Lung and bronchus	1,111,987	841,895	75.7	568,510	421,113	74.1	543,477	420,782	77.4
Pleura	489	362	74.0	273	207	75.8	216	155	71.8
Trachea, mediastinum and other respiratory organs	3086	2427	78.6	2106	1680	79.8	980	747	76.2
Bones and joints	17,176	14,054	81.8	9671	7986	82.6	7505	6068	80.9
Soft tissue including heart	60,381	50,436	83.5	33,745	27,929	82.8	26,636	22,507	84.5
Skin excluding basal and squamous	463,759	245,084	52.8	274,878	144,835	52.7	188,881	100,249	53.1
Melanoma of the skin	430,808	224,051	52.0	254,565	132,045	51.9	176,243	92,006	52.2
Other non-epithelial skin	32,951	21,033	63.8	20,313	12,790	63.0	12,638	8243	65.2
Breast, *in situ*	NA						283,751	233,502	82.3
Breast, malignant	1,294,951	1,060,064	81.9	11,236	9735	86.6	1,283,715	1,050,329	81.8
Female genital system	NA						514,641	432,279	83.9
Cervix uteri	NA						64,810	52,943	81.7
Corpus and uterus, NOS	NA						292,506	247,649	84.7
Ovary	NA						102,157	84,872	83.1
Vagina	NA						6784	5170	76.2
Vulva	NA						27,782	22,834	82.2
Other female genital organs	NA						20,602	18,811	91.3
Male genital system	NA			1,146,461	704,569	61.5	NA		
Prostate	NA			1,091,626	665,462	61.0	NA		
Testis	NA			45,227	32,402	71.6	NA		
Penis	NA			7592	5439	71.6	NA		
Other male genital organs	NA			2016	1266	62.8	NA		
Urinary system	736,493	545,604	74.1	517,044	379,579	73.4	219,449	166,025	75.7
Urinary bladder	381,247	266,866	70.0	290,764	202,408	69.6	90,483	64,458	71.2
Kidney and renal pelvis	337,171	264,252	78.4	214,718	167,923	78.2	122,453	96,329	78.7
Ureter	10,720	8935	83.3	6604	5561	84.2	4116	3374	82.0
Other urinary organs	7355	5551	75.5	4958	3687	74.4	2397	1864	77.8
Eye and orbit	15,541	11,901	76.6	8360	6336	75.8	7181	5565	77.5
Brain and other nervous system, benign	224,893	173,036	76.9	69,734	53,856	77.2	155,159	119,180	76.8
Brain, benign	10,829	8112	74.9	5081	3864	76.0	5748	4248	73.9
Cranial nerves, other nervous system, benign	214,064	164,924	77.0	64,653	49,992	77.3	149,411	114,932	76.9
Brain and other nervous system borderline	23,444	17,652	75.3	11,363	8511	74.9	12,081	9141	75.7
Brain, borderline	10,831	7515	69.4	5851	4088	69.9	4980	3427	68.8
Cranial nerves, other nervous system, borderline	12,613	10,137	80.4	5512	4423	80.2	7101	5714	80.5
Brain and other nervous system, malignant	116,569	100,037	85.8	65,525	56,741	86.6	51,044	43,296	84.8
Brain, malignant	110,062	95,140	86.4	62,282	54,256	87.1	47,780	40,884	85.6
Cranial nerves, other nervous system, malignant	6507	4897	75.3	3243	2485	76.6	3264	2412	73.9
Endocrine system	243,327	196,182	80.6	68,677	55,919	81.4	174,650	140,263	80.3
Thyroid	228,738	184,589	80.7	61,039	49,845	81.7	167,699	134,744	80.3
Other endocrine including thymus	14,589	11,593	79.5	7638	6074	79.5	6951	5519	79.4
Lymphoma	404,391	285,779	70.7	223,341	156,882	70.2	181,050	128,897	71.2
Hodgkin lymphoma	42,843	33,108	77.3	23,562	18,130	76.9	19,281	14,978	77.7
Non-Hodgkin lymphoma	361,548	252,671	69.9	199,779	138,752	69.5	161,769	113,919	70.4
Myeloma	140,054	100,911	72.1	77,923	55,985	71.8	62,131	44,926	72.3
Leukemia	264,670	173,955	65.7	154,654	101,013	65.3	110,016	72,942	66.3
Lymphocytic leukemia	127,298	76,132	59.8	77,380	46,338	59.9	49,918	29,794	59.7
Myeloid and monocytic leukemia	122,520	90,476	73.8	69,220	50,670	73.2	53,300	39,806	74.7
Other leukemia	14,852	7347	49.5	8054	4005	49.7	6798	3342	49.2
Mesothelioma	15,187	12,046	79.3	11,136	8670	77.9	4051	3376	83.3
Kaposi sarcoma	5330	3318	62.3	4821	3056	63.4	509	262	51.5

https://seer.cancer.gov/siterecode/icdo3_dwhoheme/index.html

*USCS* United States Cancer Statistics, *NCDB* National Cancer Database; *NA* not applicable; *NOS* not otherwise specified

^a^Totals include all breast disease, both males and females, miscellaneous primaries, and invalid primaries not defined in the SEER site recode ICD-O 3/WHO 2008 definitions not shown in the table.

**Table 3:** Comparison of incidences for completeness by patient demographics in 2016–2020

	USCS count	NCDB count	Case coverage (%)
*Diagnosis year*			
2016	1,835,671	1,340,154	73.0
2017	1,868,195	1,371,180	73.4
2018	1,888,798	1,389,910	73.6
2019	1,931,814	1,430,765	74.1
2020	1,744,964	1,296,498	74.3
*Age group (years)*			
0–19	84,061	56,090	66.7
20–44	661,256	531,721	80.4
45–54	1,051,339	837,344	79.6
55–64	2,234,851	1,714,153	76.7
65–74	2,801,072	2,047,766	73.1
75–84	1,752,985	1,223,798	69.8
≥85	683,878	417,635	61.1
*Pediatric, young adult age groups (years)*			
0–14	56,416	35,642	63.2
15–29	143,796	113,376	78.8
30–39	286,235	229,475	80.2
*Race/ethnicity*^a^			
White	7,673,661	5,252,315	68.4
Black	1,036,310	763,280	73.7
American Indian/Alaskan Native	59,068	24,224	41.0
Asian/Pacific Islander	336,216	237,810	70.7
Hispanic^b^	786,254	443,101	56.4

*USCS* United States Cancer Statistics, *NCDB* National Cancer Database

^a^White, black, American Indian/Alaskan Native, and Asian/Pacific Islander are shown regardless of Hispanic origin.

^b^Due to Hispanic origin misclassification, data for North Dakota and Wisconsin may be underestimated for any Hispanic race groups and overestimated for any non-Hispanic race groups.

**Table 4:** Comparison of incidences for completeness by patient state for all cancer sites in 2016–2020

	USCS count	NCDB count	Case coverage (%)
Alabama	142,136	92,044	64.8
Alaska	16,534	8493	51.4
Arizona	178,632	43,284	24.2
Arkansas	92,417	22,163	24.0
California	925,531	545,472	58.9
Colorado	133,685	106,901	80.0
Connecticut	113,707	109,051	95.9
Delaware	31,314	29,273	93.5
District of Columbia	15,210	12,162	80.0
Florida	719,491	440,952	61.3
Georgia	288,885	235,055	81.4
Hawaii	40,440	31,987	79.1
Idaho	48,273	31,760	65.8
Illinois	373,086	320,209	85.8
Indiana^a^	184,281	166,420	90.3
Iowa	101,525	71,108	70.0
Kansas	82,434	53,838	65.3
Kentucky	147,448	125,915	85.4
Louisiana	140,097	101,811	72.7
Maine	48,473	40,083	82.7
Maryland	173,825	137,654	79.2
Massachusetts	204,835	160,318	78.3
Michigan	295,481	230,478	78.0
Minnesota	168,322	134,805	80.1
Mississippi	88,204	66,443	75.3
Missouri	182,992	153,295	83.8
Montana	33,977	27,115	79.8
Nebraska	54,526	45,279	83.0
Nevada^a^	73,340	27,130	37.0
New Hampshire	46,420	39,366	84.8
New Jersey	286,034	246,754	86.3
New Mexico	50,510	26,833	53.1
New York	617,261	441,331	71.5
North Carolina	314,527	257,235	81.8
North Dakota	20,603	20,376	98.9
Ohio	362,198	323,061	89.2
Oklahoma	107,891	67,105	62.2
Oregon	117,334	88,899	75.8
Pennsylvania	422,345	356,727	84.5
Rhode Island	33,528	28,437	84.8
South Carolina	149,771	115,465	77.1
South Dakota	25,878	18,686	72.2
Tennessee	202,099	165,499	81.9
Texas	641,500	409,066	63.8
Utah	63,052	43,483	69.0
Vermont	20,649	211,524	87.6
Virginia	220,387	18,093	96.0
Washington	206,138	169,238	82.1
West Virginia	63,733	52,584	82.5
Wisconsin	183,331	151,654	82.7
Wyoming	15,152	6593	43.5

*USCS* United States Cancer Statistics, *NCDB* National Cancer Database

^a^These states did not meet the requirements for USCS publication criteria for diagnosis year 2020.

For timeliness, CoC standard 6.4 was assessed on the requirement for timely data submission, with compliance at 92.7% (Table 5).^13^ This standard has three components. The first criterion assesses compliance with monthly data submissions of all new and updated cancer cases.^13^ The second criterion ensures that all analytic cases are submitted to the NCDB’s annual call for data.^13^ The third criterion requires hospitals at least twice each calendar year to review the quality measures performance rates, which are affected by timeliness of data submission.^13^

**Table 5:** Program compliance with Commision on Cancer Data and Registry Quality Accreditaiton Standards, based on Commission on Cancer Accreditation Site Visits 2022 (*n* = 329 programs)

Validity and timeliness quality standards	CoC program compliance *n* (%)
Histologic verification for validity	
Standard 3.2: Evaluation of treatment services^a^	326/329 (99.1)
Standard 5.1: College of American Pathologists synoptic reporting	308/329 (93.6)
Reviews internal consistency for validity	
Standard 4.3: Cancer registry staff credentials	326/329 (99.1)
Re-abstracting, recoding, and reliability for validity	
Standard 6.1: Cancer registry quality control	310/329 (94.2)
Abstraction and submission timeliness	
Standard 6.4: Rapid Cancer Reporting System: data submission^b^	283/305 (92.7)

^a^Accreditation for anatomic pathology by a qualifying organization

^b^Newly accredited hospitals are not rated on standard 6.4 until their first re-accreditation visit resulting in the discrepant N.

Validity was assessed on compliance with CoC standards 3.2, 4.3, 5.1, and 6.1 at more than 90% (range, 93.6–99.1%) (Table 5). The compliance rate for CoC standard 6.1, which requires review of at least 10% of cases each year and CoC hospitals to establish a cancer registry quality control plan, was 94.2%.^13^ The re-abstracting and recoding auditing approaches involve data captured by the registry compared with data collected by a designated auditor.^11^ Compliance with histologic verification standards was high, at 93.6% for CoC standard 5.1 pathologic synoptic reporting and 99.1% for CoC standard 3.2 accreditation for anatomic pathology by a qualifying organization. The synoptic format must be structured and must include all core elements reported in a “diagnostic parameter pair” format.^13^ Each diagnostic parameter pair must be listed together in synoptic format at one location in the pathology report.^13^ Compliance with CoC standard 4.3 was at 99.1%. This standard for credentials may additionally include participation in reliability studies designed to measure abstractor and coder compliance with existing coding rules.^11^

Reproducibility is a goal in assessing the reliability study measures to help identify ambiguity or inadequacy of existing data definitions and rules as well as education needs.^11^ Edits checks at the time of data submission are part of the NCDB validity criteria and are covered in the Bray and Parkin criteria.^3^ During the 2023 annual call for data, which began in March 2023, the NCDB processed 12,151,768 records consisting of 2021 diagnoses and follow-up resubmissions from prior years. Of the total, 71,854 cases failed the NCDB edits score, representing less than 1 %.

## Discussion

The current study characterized the NCDB data quality in all four domains defined by Bray and Parkin,^3,4^ including high rates of completeness, comparability, timeliness, and validity. The cancer registry stakeholder community, demonstrated in Fig. 1 collaborates to standardize abstraction practice with universal coding definitions. The CoC accreditation standards layer an additional component to quality assurance with regard to histologic verification, registry staff credentials, synoptic reports, and inclusion of submission timeliness. Altogether, nearly all framework that applies to the hospital-based NCDB, identified by the Bray and Parkin criteria, is maintained with results indicative of consistency and stability over time.

The CoC standards for data quality that we examined are associated with high compliance and are a necessary component to maintain accreditation by the CoC. Cancer hospitals of the CoC are diverse by region, patient case mix, and volume, yet still display unified adherence to compliance with metrics designed to promote high quality of data.

Many of the countries that previously reported on national registry data quality have universal health care coverage with a single or two-tiered national provider.^5,6^ Norway has an 11-digit personal identification assigned to all newborns and people residing in the country.^5^ In contrast, the USA has a complex system of insurance options and eligibility criteria that patients navigate on their own or through their employer. The USA has no national patient identifier, and the gathering of cancer data could be further complicated by the variability in electronic health record systems, which may not be interoperable.

Despite these challenges, registrars that submit data to the NCDB demonstrate the effectiveness of quality control mechanisms developed in partnership with the registry stakeholder community, yielding high-quality data. Hospitals are required to follow standard processes and procedures to abstract and report data to the NCDB, including treatment information, and are therefore a valuable resource for evaluating cancer treatment patterns. Although central registries capture treatment information, this varies by state and therefore is not routinely available in the public facing NPCR and SEER data.

This study had limitations to be noted. First, the NCDB does not capture data beyond those hospitals accredited by the CoC. The USA has approximately 6000 hospitals,^40^ with variable definitions and practices. Through this study, we determined that the NCDB captures 73.7% of cancer patients in the USA compared with national data.

A second limitation was that the NCDB does not collect direct patient identifiers, including name. The patient’s name is necessary to run the NAACCR algorithm used by population-based registries to identify Hispanic identity, demonstrated to be of lower coverage in the NCDB.

Finally, the NCDB is not designed to assess changes in clinical practices or quality of care in real time, although with the launch of RCRS, more timely evaluation of sudden changes in cancer care and outcomes, such as those that occurred during the first months of the COVID-19 pandemic, is increasingly feasible. Mandatory concurrent data abstraction rules are in place and required of hospitals accredited by the CoC. Data submission rules are currently in place that require all new and updated cancer cases to be submitted monthly.^13^ Additional progress with timeliness is expected as the CoC standards for concurrent abstraction are adjusted to include the diagnostic and first treatment phase of care. There are plans for future studies to evaluate the completeness, comparability, validity, and timeliness of RCRS data and the feasibility of using real-time data in research.

Advances in cancer control are information dependent. As new data sources and analytic platforms become available, it is imperative that data quality be considered alongside data availability to ensure information validity and reliability. The data quality standards described in this report and adhered to by the NCDB facilitate reporting to hospital administration personnel for decision-making, researchers and epidemiologists, and quality analysts, as well as to governments that mandate reporting of cancer.

Registry data must be comprehensive, granular, and valid. High-quality data allows use of the NCDB during the CoC accreditation process to include reports on quality-of-care measures and patient outcomes assessments. The NCDB provides a comprehensive view of cancer care in the USA within CoC-accredited hospitals.
